# Colanic Acid Is a Novel Phage Receptor of *Pectobacterium carotovorum* subsp. *carotovorum* Phage POP72

**DOI:** 10.3389/fmicb.2019.00143

**Published:** 2019-02-19

**Authors:** Hyeongsoon Kim, Minsik Kim, Jaewoo Bai, Jeong-A Lim, Sunggi Heu, Sangryeol Ryu

**Affiliations:** ^1^Department of Agricultural Biotechnology, Department of Food and Animal Biotechnology, Research Institute of Agriculture and Life Sciences, Seoul National University, Seoul, South Korea; ^2^Department of Food and Nutrition, College of Human Ecology, Yonsei University, Seoul, South Korea; ^3^Research Group of Food Safety, Korea Food Research Institute, Seongnam, South Korea; ^4^Crop Cultivation and Environmental Research Division, National Institute of Crop Science, Suwon, South Korea; ^5^Center for Food and Bioconvergence, Seoul National University, Seoul, South Korea

**Keywords:** *Pectobacterium carotovorum* subsp. *carotovorum*, bacteriophage, bacteriophage receptor, colanic acid, alternative antimicrobial agent

## Abstract

The emergence and widespread nature of pathogen resistance to antibiotics and chemicals has led to the re-consideration of bacteriophages as an alternative biocontrol agent in several fields, including agriculture. In this study, we isolated and characterized a novel bacteriophage, POP72, that specifically infects *Pectobacterium carotovorum* subsp. *carotovorum* (Pcc), which frequently macerates agricultural crops. POP72 contains a 44,760 bp double-stranded DNA genome and belongs to the family *Podoviridae*. To determine the phage receptor for POP72, a random mutant library of Pcc was constructed using a Tn5 transposon and screened for resistance against POP72 infection. Most of the resistant clones had a Tn5 insertion in various genes associated with colanic acid (CA) biosynthesis. The phage adsorption rate and CA production decreased dramatically in the resistant clones. Complementation of the clones with the pUHE21-2 *lacI*^q^ vector harboring genes associated with CA biosynthesis restored their sensitivity to POP72, as well as their ability to produce CA. These results suggest that CA functions as a novel phage receptor for POP72. The application of POP72 protected Chinese cabbage from Pcc infection, suggesting that phage POP72 would be an effective alternative antimicrobial agent to protect agricultural products from Pcc.

## Introduction

*Pectobacterium carotovorum* subsp. *carotovorum* (Pcc) is a gram-negative plant pathogen causing soft rot, blackleg, and stem rot diseases in various vegetables and fruits around the world. *Pectobacterium carotovorum* subsp. *carotovorum* causes severe damage to plant tissues, and consequently, leads to dramatic drops in crop yield (Czajkowski et al., 2015), resulting in its designation as one of the top 10 most significant plant pathogens in agriculture (Mansfield et al., 2012). These bacteria produce several kinds of plant cell-wall-degrading enzymes (PCWDEs), such as pectinase, cellulase, and protease. The maceration of plant tissues during transportation and storage, as well as its growth in the field, depreciates the value of the food crop as fresh products (Lee et al., 2014).

Bacteriophages (or phages) are viruses that specifically target bacteria. The characteristics of phages, including specificity and lytic activity against host bacteria, have allowed their use in pathogen control. The relatively lower cost to produce phages than to develop novel antibiotics (Matsuzaki et al., 2005), amplification only in the living host bacteria, environmental friendliness, and a lack of side effects are additional important attractions for the widespread application of phages in diverse fields (Goodridge and Bisha, 2011). Phages infecting the various plant pathogens have been characterized and successfully applied to control various plant diseases (Czajkowski, 2016). Application of *Dikeya* phages LIMEstone1, LIMEstone2, ΦD3, and ΦD5 on potato tubers efficiently reduced *D. solani* infection (Adriaenssens et al., 2012; Czajkowski et al., 2014). Phage PP1 is the first sequenced *Podoviridae* phage of Pcc and could protect lettuce from Pcc infection (Lim et al., 2013). Other plant diseases such as fire blight caused by *Erwinia amylovora*, bacterial blotch of mushrooms caused by *Pseudomonas tolaasii*, potato infections caused by *Streptomyces scabies*, and *Ralstonia solanacearum* have also been treated with phages (Jones et al., 2007). The US Environmental Protection Agency (EPA) permitted the use of phages as a biopesticide to control bacterial spot (rot) diseases in tomatoes and peppers (United States Environmental Protection Agency (EPA), 2007; Goodridge and Bisha, 2011).

Generally, a phage has a narrow host range owing to its high specificity against a certain genus or even species of bacteria. Therefore, a mixture of various phages, designated as phage cocktail, is normally used to broaden the host range. Because the phages recognize a specific apparatus on the bacterial cell surface, such as outer-membrane proteins [e.g., FhuA, (Casjens et al., 2005)], and BtuB (Hong et al., 2008; Kim and Ryu, 2011), lipopolysaccharides, (LPS), and its component (Salgado et al., 2004; Daugelavičius et al., 2005), flagella (Kagawa et al., 1984) or pili (Yamada et al., 2007; Askora et al., 2009), bacteria often avoid phage infections by modifying or eliminating these exposed phage receptors. In this context, phage cocktails consisting of various phages utilizing different types of receptors are considered to be more effective in delaying emergence of bacterial resistance to the phage (Chan et al., 2013; Lim et al., 2013; Bai et al., 2019), suggesting that identification of phage receptor is important in phage cocktail preparation. For example, a mixture of *Salmonella* phage SSU14 recognizing O-antigens and phage SSU5 utilizing the inner core of LPS as a phage receptor efficiently delayed the growth of *S*. Typhimurium (Kim et al., 2014). However, the receptors of many phages, including Pcc- or other plant pathogen-specific phages, have not been identified, and often phage cocktails are prepared without considering the phage receptors (Wei et al., 2017; Carstens et al., 2018).

Colanic acid (CA) is one type of extracellular polysaccharides (EPS) synthesized by members of the family *Enterobacteriaceae* such as *Escherichia coli* and Pcc (Stevenson et al., 1996). CA is composed of several different types of sugars (e.g., l-fucose, d-glucose, d-galactose, and d-glucuronic acid in *E. coli*) that forms a protective capsule surrounding the bacterial cell surface (Andrianopoulos et al., 1998). These capsular EPS play a critical role in survival of bacteria under various environmental stresses, such as dehydration and osmotic shock (Gottesman and Stout, 1991; Stevenson et al., 1996). EPS are also virulence factors in many enterobacteria, for example, bacterial adherence and biofilm formation are affected by the CA in *Salmonella* and *E. coli* (Danese et al., 2000; Solano et al., 2002; Hanna et al., 2003; Matthysse et al., 2008) and amylovoran is one of major virulence factors of *E. amylovora* (Piqué et al., 2015). Capsular EPS are recognized as a receptor by several phages; Vi-antigen of *Salmonella*, K-antigen of *E. coli*, and amylovoran of *E*. *amylovora* have been characterized as phage receptors (Gross et al., 1977; Pickard et al., 2010; Roach et al., 2013). However, phages that recognize CA as a receptor have not been reported.

In this study, we identified the CA of Pcc as a novel phage receptor for phage POP72. POP72 effectively protected the Chinese cabbage from Pcc infection, suggesting that it could be developed as a promising alternative antimicrobial agent to protect crops against Pcc.

## Materials and Methods

### Bacterial Strains and Growth Conditions

The bacterial strains used in this study are listed in Table 1. *Pectobacterium carotovorum* subsp. *carotovorum* (Pcc) isolates were kindly provided by the Rural Development Administration (RDA) at Wanju-gun, Korea. All the Pcc isolates were incubated in tryptic soy broth (TSB) and tryptic soy agar (TSA) [1.5% (w/v) agar] at 28°C. *Escherichia coli* MFD*pir* was grown in Luria-Bertani (LB) broth and plates supplemented with 0.3 mM diaminopimelic acid (DAP). Antibiotics were added to the media at the following concentrations if necessary: ampicillin (Amp), 50 μg mL^−1^; carbenicillin (Car), 100 μg mL^−1^; kanamycin (Kan), 50 μg mL^−1^; rifampicin (Rif), 50 μg mL^−1^, while isopropyl β-D-1-thiogalactopyranoside (IPTG) was added at a concentration of 50–100 μM.

**Table 1:** Bacterial strains and plasmids used in this study.

**Strain and plasmid**	**Genotype and main characteristics^a^**	**References**
***Pectobacterium carotovorum*** **subsp**. ***caorotovorum***		
Pcc27	*Pectobacterium carotovorum* subsp. *carotovorum* isolate Pcc27; Wild type; host for phage POP72	RDA^b^
Pcc27^RifR^	Spontaneous rifampicin-resistant mutant of Pcc27	This study
Pcc27 + pUHE21-2	Pcc27 with pUHE21-2 *lacI*^q^	This study
Pcc27^Mu−1^	Spontaneous POP72-resistant mutant of Pcc27	This study
Pcc27^Mu−2^	Spontaneous POP72-resistant mutant of Pcc27	This study
*cpsG*::Tn5	Pcc27 with transposon insertion in putative *cpsG*	This study
*wcaA*::Tn5	Pcc27 with transposon insertion in putative *wcaA*	This study
*wzc*::Tn5	Pcc27 with transposon insertion in putative *wzc*	This study
*cpsG*::Tn5 + pUHE21-2	*cpsG*::Tn5 with pUHE21-2 *lacI*^q^	This study
*cpsG*::Tn5 + pCpsG	*cpsG*::Tn5 complementation with homologous *cpsG* gene from Pcc21	This study
*wcaA*::Tn5 + pUHE21-2	*wcaA*::Tn5 with pUHE21-2 *lacI*^q^	This study
*wcaA*::Tn5 + pWcaA	*wcaA*::Tn5 complementation with homologous *wcaA* gene from Pcc21	This study
*wzc*::Tn5 + pUHE21-2	*wzc*::Tn5 with pUHE21-2 *lacI*^q^	This study
*wzc*::Tn5 + pWzc	*wzc*::Tn5 complemented with WT *wzc* gene	This study
Pcc27^Mu−1^ + pUHE21-2	Pcc27^Mu−1^ with pUHE21-2 *lacI*^q^	This study
Pcc27^Mu−1^ + pWzc	Pcc27^Mu−1^ complemented with WT *wzc* gene	This study
Pcc27^Mu−1^ + pWzc^Mu−1^	Pcc27^Mu−1^ complemented with *wzc*^Mu^ gene from Pcc27^Mu−1^	This study
Δ*wzc*	*wzc* in-frame deletion mutant of Pcc27	This study
***Escherichia coli***		
DH5α λ*pir*	Φ80 *lacZ*ΔM15 Δ(*lacZYA-argF*) U169 *recA1 hsdR17 endA1 deoR thi-1 supE44 gyrA96 relA1*/λ*pir*	Platt et al., 2000
MFD*pir*	MG1655 RP4-2-Tc::[ΔMu1::Δ*aac(3)IV*-Δ*aphA*-Δ*nic35*-ΔMu2::*zeo*] Δ*dapA*::(*erm*-*pir*) Δ*recA*	Ferrieres et al., 2010
**Plasmids**		
pUHE21-2 *lacI*^q^	rep_pMB1_ *lacI*^q^; inducible Lac promoter; Amp^R^	Soncini et al., 1995
pCpsG	pUHE21-2 *lacI*^q::^*PCC21_RS06735*; Amp^R^	This study
pWcaA	pUHE21-2 *lacI*^q^::*PCC21_RS06680*; Amp^R^	This study
pWzc	pUHE21-2 *lacI*^q^::*wzc*; Amp^R^	This study
pWzc^Mu−1^	pUHE21-2 *lacI*^q^::*wzc*^Mu−1^; Amp^R^	This study
pKD46	*repA101*(ts) *oriR101 bla* P_araB_-(*gam bet exo*); Amp^R^	Datsenko and Wanner, 2000
pKD13	*oriR_γ*R*6*k*_ bla FRT::kan::FRT*;Kan^R^	Datsenko and Wanner, 2000
pCP20	λ*cI857*(ts) *repA101*(ts) *oriR101 bla cat* λ*p*_R_-FLP;Amp^R^, Cm^R^	Datsenko and Wanner, 2000

a Amp^R^, ampicillin resistant, Kan^R^, kanamycin resistant, Cm^R^, chloramphenicol resistant.

b *RDA, the Rural Develop Administration (Wanju-gun, South Korea)*.

### Phage Spot Assay

Bacterial susceptibility against the phage was confirmed with the spot assay as described previously (Kim and Ryu, 2012). Briefly, the host bacterial lawn on the TSA plate was prepared by pouring 5 mL of soft TSB agar [0.4% agar (supplemented with antibiotics and IPTG, if necessary)] inoculated with the host bacteria. After 30 min of solidification, 10 μL of serially diluted (10-fold) phage lysates were spotted on the bacterial lawn and dried for 20 min at room temperature. Plates were incubated overnight at 28°C, and the phage plaques formed were counted on the next day.

### Transmission Electron Microscopic Analysis

Purified phage POP72 was morphologically characterized by transmission electron microscopic (TEM) analysis as described by Kim and Ryu (2011). Briefly, a high titer of phage stock (~1 × 10^10^ PFU mL^−1^) was adsorbed onto carbon-coated copper grids and negatively stained with 2% aqueous uranyl acetate (pH 4.0). The phages were observed with TEM (Carl Zeiss LEO 913AB) at 100-kv accelerating voltage at the National Institute of Agricultural Sciences (Wanju-gun, South Korea). The phage was classified morphologically using the International Committee on Taxonomy of Viruses (ICTV) classification (King et al., 2011).

### Sequencing of POP72 Genomic DNA and Genome Analysis

Phage DNA was extracted from the virions with proteinase K and SDS as previously described (Sambrook and Russell, 2001). The purified phage DNA was sequenced with a Genome Sequencer FLX titanium sequencer (Roche, Mannheim, Germany) and assembled with GS *de novo* assembler software (Roche) at Labgenomics Inc., South Korea. The ORFs of the POP72 genome were predicted using Glimmer3 (Delcher et al., 1999), Genemark.hmm (Lukashin and Borodovsky, 1998), FgenesB software (Softberry, Inc., Mount Kisco, NY), and RAST annotation engine (http:://rast.nmpdr.org/) (Aziz et al., 2008; Overbeek et al., 2014). The assortment and editing of genome-sequencing and annotation data were conducted using Artemis (Carver et al., 2008). The tRNA-coding sequences in the phage genome were scanned with a tRNAscan-SE program (Lowe and Eddy, 1997). The prediction of protein functions was conducted with NCBI BLASTp, CDD (Conserved Domain Database), and InterProscan (Altschul et al., 1990; Jones et al., 2014; Marchler-Bauer et al., 2014). A comparative genomic analysis of POP72 with homologous phages was conducted by BLASTn, and the results were visualized using ACT13 (Carver et al., 2008). Putative promoters and terminators in CA biosynthesis gene cluster were predicted using BPROM and iTerm-PseKNC (Solovyev and Salamov, 2011; Feng et al., 2018). BioCyc database collection was used to predict putative operons from the gene cluster (Karp et al., 2017).

### Construction of a Tn5 Transposon Mutant Library and Screening Phage Resistance Mutants

A Tn5 transposon mutant library was constructed as previously described (Larsen et al., 2002; Ferrieres et al., 2010) with some modifications. Briefly, a donor *E. coli* MFD*pir* containing a suicide vector pRL27 and a recipient Pcc27 at early log phase (optical density at 600 nm, ~0.8) was harvested and washed three times with 10 mM MgSO_4_. A mixture of donor and recipient cells (3:1, v/v) was spotted onto the TSA plate supplemented with DAP and incubated for 24 h at 28°C for the conjugation. To screen the phage-resistant mutants, pools of transconjugants were mixed with POP72 (10^9^ PFU mL^−1^) in 1 mL of TSB broth and incubated at room temperature for 15 min prior to plating on TSA/Kan plates for 24 h at 28°C. Surviving colonies were isolated by streaking three times on TSA plates, and the absence of the remaining phages in the cells was verified by spotting culture supernatants on the bacterial lawn. Isolated phage-resistant Tn5 mutants were further characterized.

### Determination of the Transposon Insertion Site by Rescue Cloning

Genomic DNA extracted from the selected transposon mutants was digested with the restriction enzyme *Bam*HI, and purified DNA fragments were self-ligated with T4 ligase (Roche). *E. coli* DH5α λ*pir* was transformed with the circularized DNA fragments by heat shock and plated on LBA/Kan. Rescued plasmid DNA harboring the R6Kγ origin with the Tn5 transposon was extracted from the selected clones, and a locus of transposon insertion was identified by DNA sequencing using a pair of oligonucleotide primers tpnRL17-1 and tpnRL 13-2 (Table 2) (Larsen et al., 2002). The nucleotide sequence of *Pectobacterium carotovorum* subsp. *carotovorum* strain Pcc21 (GenBank accession number CP003776) that was registered in the NCBI database was used as a reference for the sequence analysis.

**Table 2:** Primers used in this study.

**Primer**	**Sequence (5^**′**^-3^**′**^)**
**CONSTRUCTION OF PLASMID**	
Pcc27_*cpsG*_F_BamHI	ATAGGATCCGGCTAAGCGCTGTTGCAGGAAAG
Pcc27_*cpsG*_R_HindIII	ATAAAGCTTGAGAAGGTAATAGACGATACTGAA
Pcc27_*wcaA*_F_BamHI	ATAGGATCCATGTCAACAAATAATTTAGTCAGTGTTATTATT
Pcc27_*wcaA*_R_HindIII	ATAAAGCTTTGAACGCAAGTCAATCATTTTATTTTTTCC
Pcc27_*wzc*_F_BamHI	ATAGGATCCAGCAATCAGCTCAGAAGTGGGCA
Pcc27_*wzc*_R_HindIII	ATAAAGCTTATGCGAACATCCGGTTATCACAAGG
**SEQUENCE CONFIRMATION**	
Pcc27_RS06635_F_confirm	GATACGGGGAACTGGGGGCCTTTT
Pcc27_ RS06635_F1	CCCCCTCAAAATGGATCGTAG
Pcc27_ RS06635_F3	GGCGAAGATCCTGAACTAACC
Pcc27_ RS06635_F4	CGTGTCGCTGCTATGCCAAAA
Pcc27_ RS06635_F5	AGCCCGGCAATTGGTAAAACG
Pcc27_ RS06635_F6	CTGGCTGTAACCGATGCTGCT
Pcc27_ RS06645_R_confirm	TAAGGAAGACCGGATGCGAACATCC
Pcc27_ RS06645_R1	AGCAACCGCTTTTGAACCACG
Pcc27_ RS06640_F_confirm	AGAGTCTTATTATGCAGAGTGCT
Pcc27_ RS06645_R_confirm	GCGGGGAGATGGGTAACAAA
Pcc27_ RS06645_F_confirm	GTTACGATAGGTCAAGGCGC
Pcc27_ RS06640_F1	TTGCATTCTTCCAACATCTCGC
Pcc27_ RS06640_F2	GTTAAGTCCATAGTGCCTTGGCT
Pcc27_ RS06640_F3	CGGCATGTGTTAGGTAGTGATG
Pcc27_ RS06640_ R1	CCAGTTTGAGATCTCTGGGCA
Pcc27_ RS06640_R2	AGAGGGATGTTGGATTGGGGC
Pcc27_ RS06640_R3	ATTACCGTAAACACCACCGCC
Pcc27_RS06725_F_confirm	CATGAACGTCTGGACTTGACAACAA
Pcc27_RS06735_R_confirm	TTCAGTACCGGATAAACCGATGTCA
Pcc27_RS06675_F_confirm	CCTATCGCGTTAATTGCTGTAC
Pcc27_RS06685_R_confirm	GCATTGCATTCTCCCAACATCT
pUHE21-2_F1	AGATTCAATTGTGAGCGGATAAC
pUHE21-2_R3	GGTCATTACTGGATCTATCAACA
tpnRL17–1	AACAAGCCAGGGATGTAACG
tpnRL13–2	CAGCAACACCTTCTTCACGA
**CONSTRUCTION OF IN-FRAME DELETION MUTANT**	
Pcc27_wzc_60bp_F_pKD13	TCAGCTCAGAAGTGGGCACAAGCATTAAGCCGTTAATAAGTCAG TGTAGGCTGGAGCTGCTTCG
Pcc27_wzc_60bp_R_pKD13	GCTGAATAGCAGAGGGATCAATTATACGAACATTACCCACTGTACTATTCCGGGGATCCGTCGACC

### Construction of In-frame Deletion Mutant in *Pectobacterium*

Site-directed mutagenesis of Pcc27 was performed by the lambda red recombination system with some modifications (Datsenko and Wanner, 2000). Fifty milliliter of Pcc27 cells harboring plasmid pKD46 were incubated at OD_600_ = 0.5 and made electrocompetent by three time washes with ice-cold 10% glycerol. The kanamycin resistant (Kan^R^) cassette from plasmid pKD13 was amplified using primers that contains 40-mer homologous sequences of *wzc* gene and 20-mer priming sequence of pKD13. Fifty microliter of the competent cells were transformed with 3.5 μg of the concentrated PCR product by electroporation, and recovered in 1 ml SOC media for 3 h at 30°C. Two-hundred microliter of the culture were plated out on LBA/Kan. The remaining cell cultures were kept at room temperature overnight and plated out the next day. The Kan^R^ cassette of the positive transformants was removed by introducing the plasmid pCP20. Gene deletion was confirmed by PCR and subsequent DNA sequencing.

### Isolation of Spontaneous POP72-Resistant Pcc27 Mutants

To isolate spontaneous POP72-resistant mutants, a high-titer overlay assay was performed as previously described (Kim and Ryu, 2011). Briefly, 5 mL of soft TSB agar (0.4% agar) inoculated with a mixture of serially diluted (10-fold) Pcc27 culture at stationary phase and 100 μL of POP72 suspension (~10^9^ PFU mL^−1^) were poured on TSA plates. The solidified plates were incubated for 24 h at 28°C. Randomly selected single colonies from each plate were isolated by repeated subcultures on TSA plates. The phage resistance and the absence of phages from the isolates were verified using a spot assay.

### Plasmid Construction

The plasmids used in this study are listed in Table 1. Plasmid pCpsG, which expresses the putative *cpsG* gene under the control of the *lac* promoter, was constructed as follows. The putative *cpsG* gene from Pcc27 was amplified by PCR with oligonucleotides Pcc27_*cpsG*_F_*Bam*HI and Pcc27_*cpsG*_R_*Hin*dIII. The purified PCR product was digested with the restriction enzymes *Bam*HI and *Hin*dIII and ligated with the same restriction enzyme-digested pUHE21-2 *lacI*^q^ plasmid vector (Soncini et al., 1995). The sequence of the cloned gene was verified by DNA sequencing. All the other plasmids were constructed with similar experimental steps and corresponding oligonucleotides (Table 2).

### Phage Adsorption Assay

The phage adsorption assay was performed as previously described with some modifications (Kim and Ryu, 2012). Briefly, Pcc cells were harvested and suspended in TSB media (approximately 2~3 × 10^8^ CFU mL^−1^). Phage POP72 was added to the bacterial suspension at an MOI of 0.01 and aliquoted in an equal volume to five microtubes. During the phage adsorption for 15 min at 28°C, each tube was centrifuged (16,000 × g, 4°C for 1 min) at the indicated time points, and the supernatants were immediately filtered (0.22 μm-pore-size filter). The number of remaining free phage particles in the filtrates was determined by the spot assay using Pcc27 as an indicator strain.

### Extraction and Quantification of CA

The CA of Pcc was extracted as previously described with some modifications (Dische and Shettles, 1951). EPS-degrading enzymes in the Pcc cell culture were inactivated by heating at 100°C for 15 min. After cooling, 50 mL of the culture were centrifuged (13,000 × g, 4°C for 30 min), and the supernatant was mixed with three volumes of ethanol. CA-containing precipitates were harvested by centrifugation as described above after overnight incubation at 4°C. The pellet was dissolved in 4 mL of distilled water and dialyzed for 48 h against distilled water (Spectra/Por 3 regenerated cellulose membrane, MWCO 3.5 kDa, SPECTRUM). Residual polypeptides were removed by precipitation with 5 mL of 10% (v/v) trichloroacetic acid for 1 h and centrifuged (13,000 × g, 4°C for 30 min). The supernatant was dialyzed again for 120 h against distilled water. The resulting preparation was stored at 4°C until quantification.

The quantification of CA was conducted by measuring the amount of non-dialyzable methyl pentose (i.e., fucose) which is a specific component of the CA (Bechet et al., 2010). One milliliter of the CA preparation was mixed with 4.5 mL of H_2_SO_4_/H_2_O (6:1, v/v), and the mixture was heated at 100°C for 20 min and cooled to room temperature. For each sample, the absorbance at 396 nm (A_396_) and 427 nm (A_427_) was measured either directly (control sample, A-co) or after the addition of 100 mL of cysteine hydrochloride (cysteine sample, A-cy) (Bechet et al., 2010). Biological extracts contained various compounds, which following reaction with H_2_SO_4_, yield products absorbing between 396 and 427 nm. Nonspecific reactions were subtracted from the total absorption of the sample: the value of A_396−co_ and A_427−co_ were deducted from A_396−cy_ and A_427−cy_, respectively, to acquire ΔA_396_ and ΔA_427_. (ΔA_396_-ΔA_427_) were associated with the concentration of methylpentose using a diverse concentration of fucose standard curve ranging from 5 to 100 μg mL^−1^ (Obadia et al., 2007).

### Phage Inactivation by Extracted Pcc27 CA

Inactivation of the phage particles by Pcc27 CA was examined as previously described with modifications (Heller and Braun, 1979). Briefly, dilutions of phage POP72 (5 × 10^6^ PFU mL^−1^) were mixed with equal volumes of extracted Pcc27 CA (36 μg mL^−1^) and incubated at 28°C for 30 min. The mixtures were diluted 10-fold with ice-cold PBS, and the number of remaining active phage particles was counted using the double-agar layer overlay assay. The number of phages from a parallel assay conducted with distilled deionized water (ddH_2_O) was used as a negative control.

### Isolation of Spontaneous Rifampicin-Resistant Pcc27 Mutants

Rifampicin-resistant mutants were selected as previously described (Glandorf et al., 1992). Spontaneous rifampicin-resistant mutants of Pcc27 were obtained by transferring colonies of this strain to TSA agar plates containing increasing concentrations (12.5, 25, 37.5, and 50 μg mL^−1^) of rifampicin (Sigma). The stability of these rifampicin-resistant mutants was tested by three subcultures of the rifampicin-resistant mutants on TSA agar plates containing rifampicin (50 μg mL^−1^).

### Pcc Virulence Assay in Chinese Cabbage

A Pcc virulence assay was performed as previously described with modifications (Marquez-Villavicencio et al., 2011). Chinese cabbages purchased in a local market in Seoul, South Korea were cut to an equal size (~10 by 7 cm) and surface sanitized for 5 min with 1% sodium hypochlorite. After rinsing with sterilized water three times for 5 min and air-drying, Chinese cabbage samples were stabbed with a sterilized pipette tip, and 10 μL of Pcc bacterial culture (10^9^ CFU mL^−1^) was added to the wound. The negative control was inoculated with sterile distilled water. For the phage-treated group, POP72 lysate was placed on the Pcc-inoculated wound at an MOI of 1, 10, and 100. The Chinese cabbages were placed in plastic boxes to maintain high humidity and incubated at 28°C for 24 h. The samples were examined periodically for soft rot disease symptoms. The number of Pcc recovered from the Chinese cabbage was measured as described by Lee et al. (2016). Rifampicin-resistant Pcc27 (Pcc27^RifR^) cells from the inoculated Chinese cabbage samples were separated and collected using stomaching with 100 mL of sterilized buffered peptone water (B.P.W) for 1 min. After the stomaching, the Chinese cabbage debris was removed using a sterile bag (FILTRA-BAG with Open Top, SCTO7012A, LABPLAS), and the supernatants were transferred to new conical tubes. After centrifugation at 13,000 × g, 4°C for 1 min, the bacterial cell pellets were resuspended with PBS buffer. Serially diluted (10-fold) cell suspensions were spread onto LB Rif agar plates, and the colonies formed were counted after overnight incubation at 28°C.

## Results and Discussion

### Isolation and Characterization of Phage POP72

*Pectobacterium carotovorum* phages were screened by a plaque assay from environmental samples (i.e., soil from cultivated land, agricultural water, and irrigation water) collected from various regions in South Korea. Nine phages were isolated, and the ability of these phages to infect bacteria was confirmed by single plaque formation in a spot assay with serially diluted phage lysate (10-fold; from 10^9^ to 10^2^ PFU/ml). Phage POP72 has the broadest host range compared with the other eight phages and forms clear plaques on several Pcc (20 of 22 strains) and *P. carotovorum* subsp. *brasiliensis* strains (Pcb; 13 of 15 strains) (Table S1). Morphologically, POP72 belonged to the family *Podoviridae* with an icosahedral head (60.09 ± 2.79 nm) and a short tail (15.00 ± 3.80 nm) (Figure S1).

Genomic analysis revealed that POP72 has 44,760 bp of double-stranded DNA with a GC content of 49.7% containing 55 putative open reading frames (ORFs) and no tRNAs (Figure S2 and Table S2). No genes known to be associated with lysogen formation were found, suggesting the lytic nature of the POP72. Comparative genomic analysis using BLAST and ACT13 revealed that phage POP72 highly resembled *Pectobacterium carotovorum* phage PP1 (Lim et al., 2013). Although the whole genome of phage POP72 shares 99% nucleotide identity with the PP1 genome (Figure S3), an additional 360-bp sequence flanked by ORF28 (endonuclease) and ORF29 (phosphoesterase) exists in POP72. With the exception of phage PP1, phage phD2B that specifically infects the plant pathogen *Lelliottia* spp. is the phage most similar to POP72 (Figure S3).

### Identification of the Phage Receptor for Phage POP72

**Figure 1:**
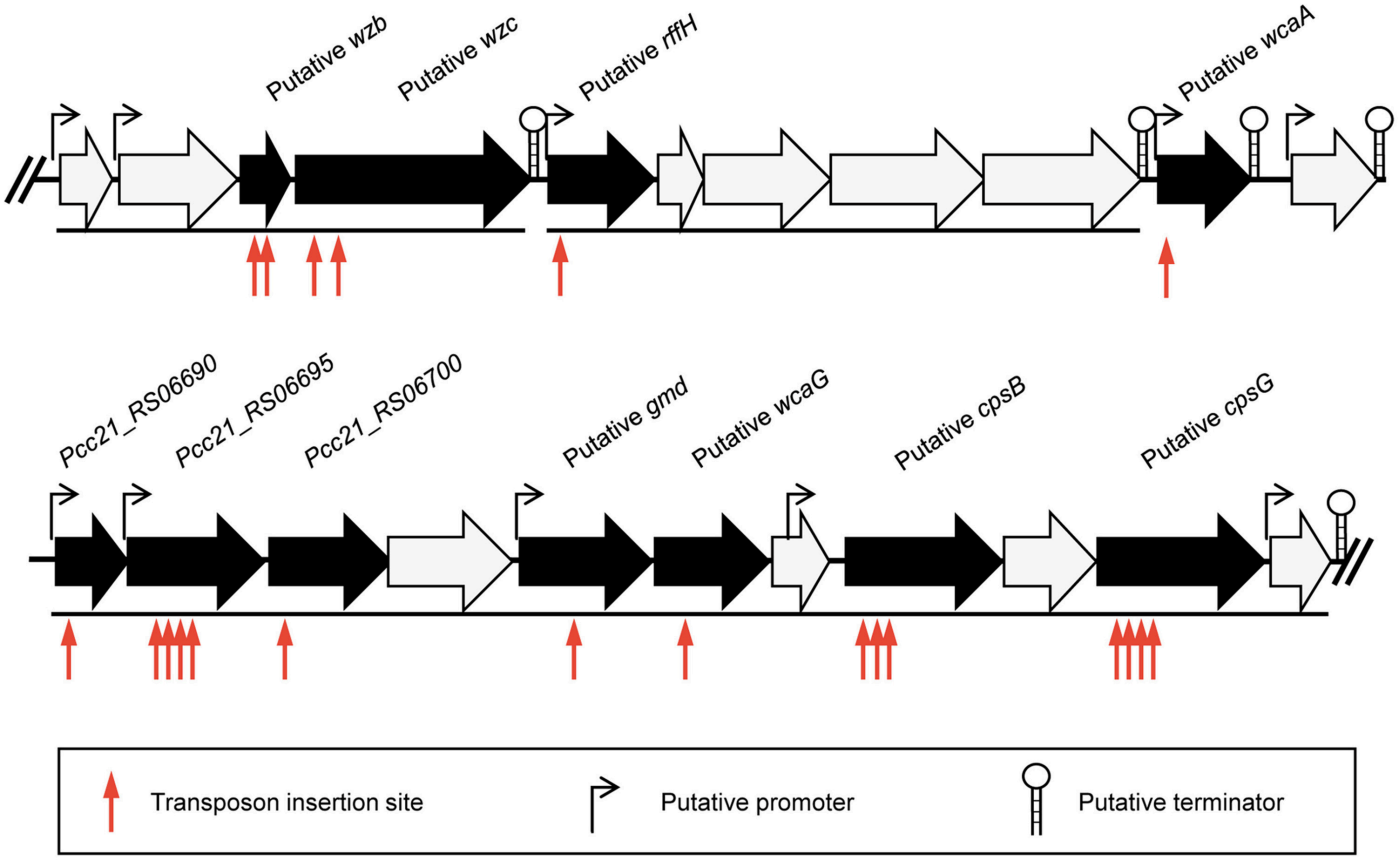
Schematic representation of the predicted gene cluster for CA biosynthesis in Pcc27. Black arrows indicate genes disrupted by the Tn5 transposon insertion and black lines under the genes indicate the predicted operons. The predicted promoters and terminators are also shown.

To identify the phage receptor for POP72, a random mutant library of *Pectobacterium carotovorum* subsp. *carotovorum* isolate Pcc27 was constructed using a Tn5 transposon (Larsen et al., 2002; Ferrieres et al., 2010), and POP72-resistant clones were screened. Twenty-four clones of POP72-resistant Pcc27 were obtained from the library (Table 3). Through partial DNA sequencing, 21 of the POP72-resistant clones were found to have transposon insertions in various genes associated with CA biosynthesis (Figure 1), including operons and genes involved in the exportation of CA repeat unit to periplasm, biosynthesis of polysaccharides, and biosynthesis of precursor sugar of CA. Among these, three putative genes, putative *cpsG* (homology to *Pcc21_RS06735* in *Pectobacterium carotovorum* subsp. *carotovorum* strain Pcc21), putative *wcaA* (*Pcc21_RS06680* in Pcc21), and putative *wzc* (*Pcc21_RS06645* in Pcc21), were selected for further investigation. The *cpsG* gene in the other *Enterobacteriaceae* bacteria is directly involved in the biosynthesis of GDP-mannose, which is converted into a GDP-fucose via a three-step pathway (Andrianopoulos et al., 1998). Two l-fucoses and one d-glucose constitute a main chain of CA monomer, and a side chain comprised of d-galactose and d-glucuronic acid is attached to the one of l-fucoses, suggesting that sufficient expression of the *cpsG* gene may be required to construct a basic structure of CA (Stevenson et al., 1996). The putative *wcaA* and putative *wzc* genes encode a glycosyltransferase and an exopolysaccharide exporter accessory protein, respectively. Although the exact function of WcaA glycosyltransferase has not been clearly revealed, this enzyme might be associated with the transfer of some saccharide moieties to biosynthesize the CA. The gene product of the putative *wzc* might play a role in exporting the capsular polysaccharide beyond the outer membrane and regulating the tyrosine phosphorylation of the UDP-glucose dehydrogenase involved in the CA biosynthesis (Table 3) (Stevenson et al., 1996; Grangeasse et al., 2003; Bechet et al., 2010).

**Table 3:** List of genes disrupted by Tn5 insertion.

**Putative gene**	**Locus_tag of homologous gene in Pcc21^a^**	**Putative protein**	**Number of Tn5 mutant**
*wzb*^b^, ^c^	Pcc21_RS06640	Protein-tyrosine-phosphatase	2
*wzc*^b^, ^c^	Pcc21_RS06645	Exopolysaccharide exporter accessory protein; tyrosine-protein kinase	2
*rffH*^d^	Pcc21_RS06650	Glucose-1-phosphate thymidylyltransferase	1
*wcaA*^b^	Pcc21_RS06680	Glycosyl transferase family 2	1
N/A^b^	Pcc21_RS06690	O-acetyltransferase	1
*wca* operon^b^	Pcc21_RS06695	Glycosyl transferase family 1	4
*wca* operon^b^	Pcc21_RS06700	Glycosyl transferase	1
*gmd*^b^	Pcc21_RS06710	GDP-mannose 4,6-dehydratase	1
*wcaG*^b^	Pcc21_RS06715	GDP-fucose synthetase	1
*cpsB*^b^, ^c^	Pcc21_RS06725	Mannose-1-phosphate guanylyltransferase	3
*cpsG*^b^, ^c^	Pcc21_RS06735	Phosphomannomutase	4
*leuD*	Pcc21_RS17900	Isopropylmalate isomerase small subunit	1
N/A^e^	Pcc21_RS17920	Methyl-accepting chemotaxis protein	1
*nfuA*	Pcc21_RS19445	Fe-S biogenesis protein	1

a Homologous genes in Pcc21 were indicated for reference because the whole genome of Pcc27 was not sequenced yet.

b (Stevenson et al., 1996).

c (Cozzone et al., 2004).

d (Marolda and Valvano, 1995).

e N/A, not available.

**Figure 2:**
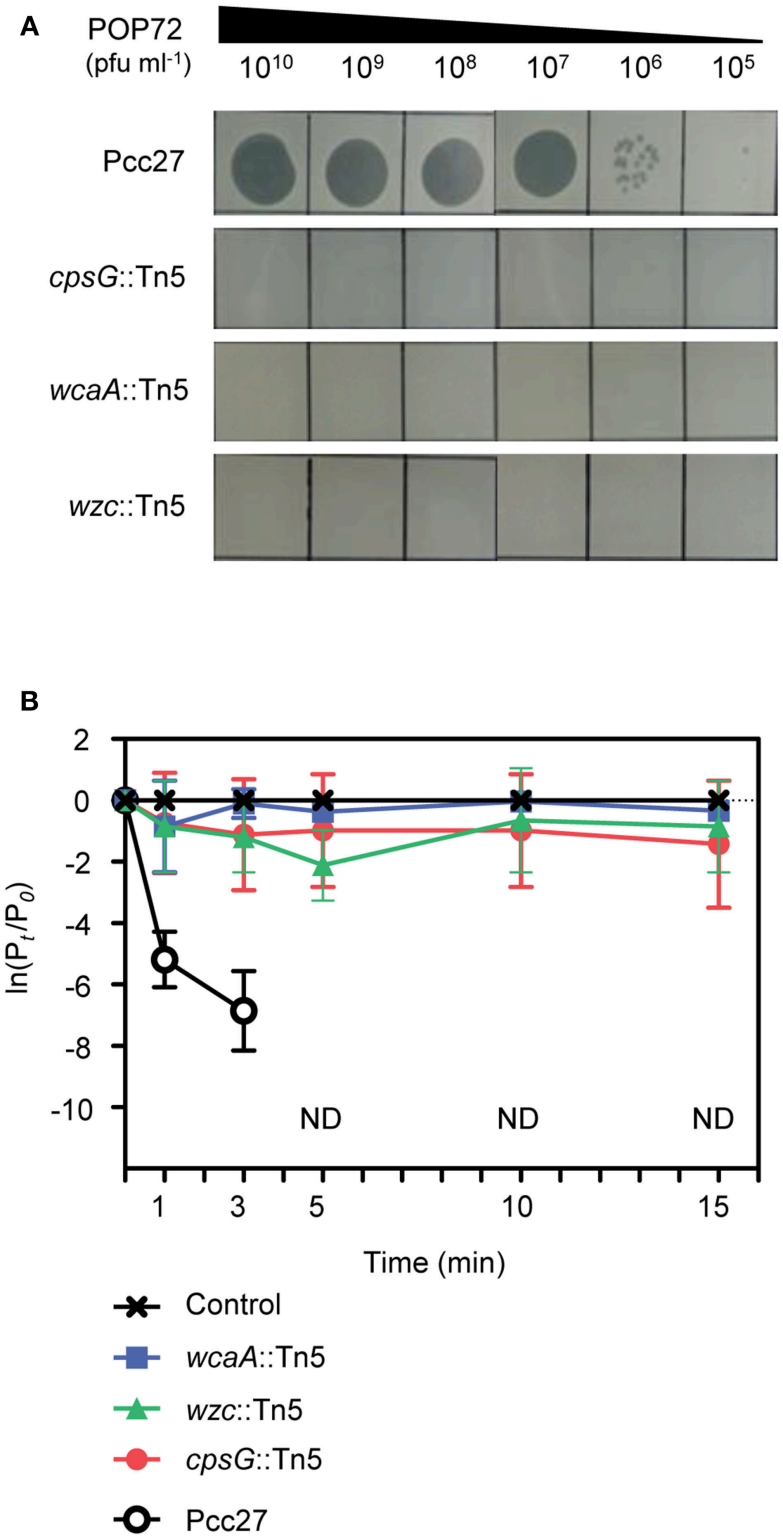
Tn5 insertions in the genes associated with the CA biosynthesis abolished the POP72 sensitivity in Pcc27. **(A)** Transposon mutants with disrupted *cpsG, wcaA*, or *wzc* genes by the Tn5 insertion did not form POP72 plaques in the phage spot assay. **(B)** The initial binding of POP72 to Pcc27 cells was abolished by the Tn5 insertion in the *cpsG, wcaA*, or *wzc* genes. Pcc27 cells in the early exponential phase were infected with POP72 (MOI = 0.01) and incubated at 28°C for the times indicated. After centrifugation and filtration, the phage titer in the filtrate was determined using a spot assay. The results are expressed as the means and standard deviations of triplicate assays. P_*t*_, phage titer (PFU mL^−1^) at time *t*; P_0_, initial phage titer; ND, not detected.

**Figure 3:**
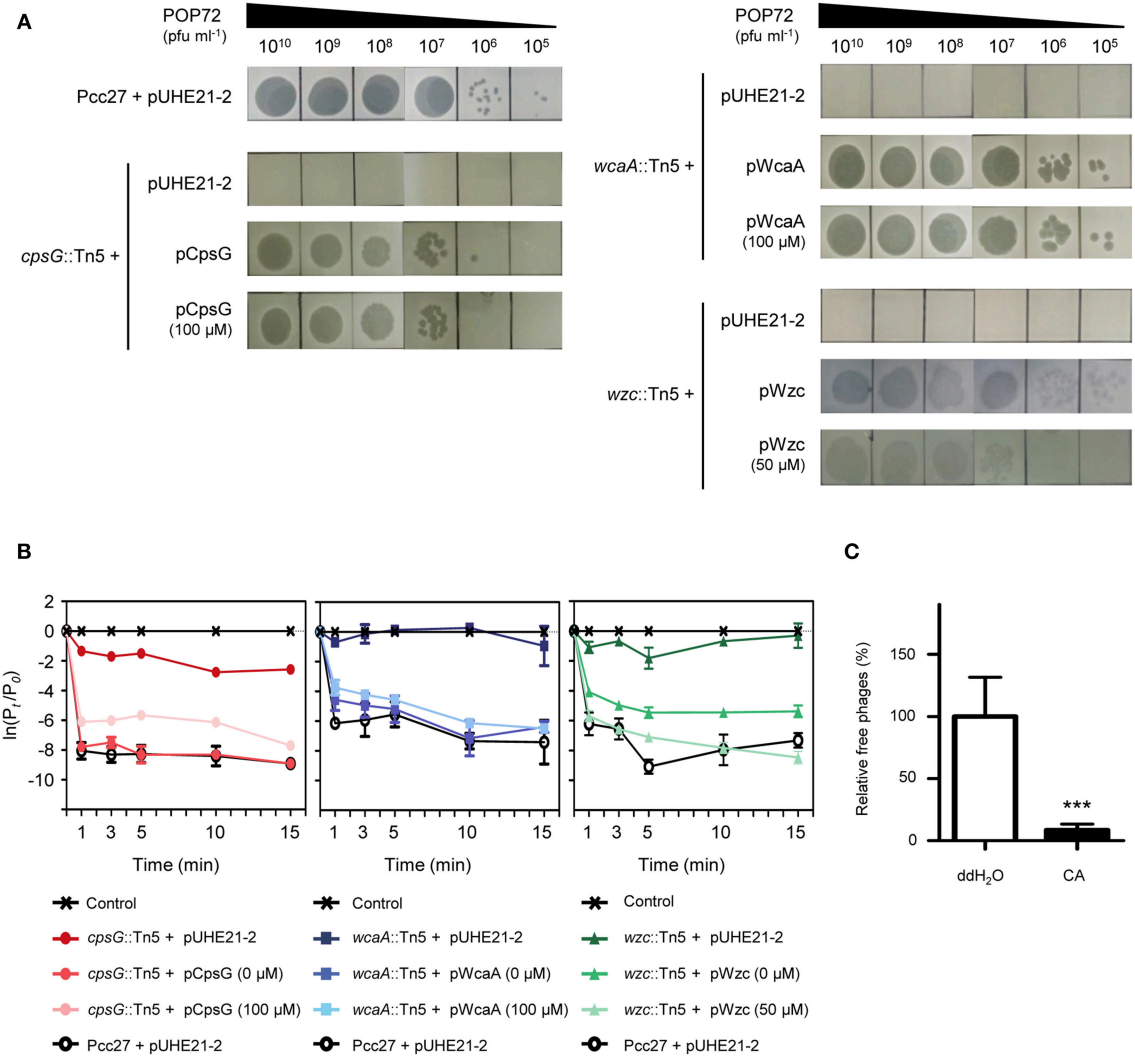
Confirmation of Pcc27 CA as a phage POP72 receptor. Complementation of the CA biosynthesis gene in Tn5 mutants partially or completely restored the phage susceptibility **(A)** and initial phage binding **(B)**. The concentration of IPTG is indicated in parentheses. **(C)** Specific interaction between phage POP72 and Pcc27 CA. Inactivation of the POP72 particles by extracted Pcc27 CA was investigated by counting the remaining phages after the co-incubation of phages and CA. An independent parallel assay with distilled deionized water (ddH_2_O) was also conducted to use as a negative control. One representative **(A)** or the means with standard deviations **(B,C)** of triplicate experiments are shown. ^***^P < 0.0001.

The susceptibility of these Tn5 transposon mutants, as well as the Pcc27 wild-type (WT) strain, against POP72 was examined further using the spot assay. As expected, phage POP72 could not form any plaques on the lawns of the three transposon mutants (Figure 2A). When the Tn5-inactivated genes were complemented by a pUHE21-2 *lacI*^*q*^ vector harboring each corresponding gene, the sensitivity of Pcc27 against POP72 was partially or completely restored even without IPTG induction (Figure 3A). Complementation without IPTG induction, as well as the partial restoration, might be due to the improper levels of each protein within a bacterial cell because it is difficult to fine-tune the expression levels of genes with the leaky inducible *lac* promoter and IPTG (Jacob and Monod, 1978). The in-frame *wzc* gene deletion mutant was also constructed and tested for the phage susceptibility. As expected, the Δ*wzc* mutant was resistant to POP72 and the *wzc* complemented strain was sensitive to POP72 (Figure S4), suggesting that insensitivity of transposon mutants against POP72 was not due to the polar effects of the Tn5 insertions on adjacent gene expression. To test whether the inactivated genes played a role in the initial step of POP72 infection, phage adsorption was assayed with the WT and Tn5 mutants. As shown in Figure 2B, most of phage POP72 efficiently bind to the WT Pcc27 cells within 1 min, and no infectious particles were remained in the supernatant after 5 min. However, the phage POP72 adsorption to the Pcc27 cells was completely abolished in the *cpsG*::Tn5*, wcaA*::Tn5, and *wzc*::Tn5 mutants. The complementation of these genes restored the POP72 adsorption in the mutants (Figure 3B). In addition, the specificity of POP72 to the CA was verified further by phage inactivation assays using the CA extracted from the WT Pcc27 strain. As shown in Figure 3C, POP72 directly interacts with the CA. Approximately 80% of the POP72 particles were inactivated by 36 μg mL^−1^ of Pcc27 CA at 28°C within 30 min. The inactivation might be due to a triggering of DNA ejection from the POP72 head upon CA binding, similar to the case of *Podoviridae* phage P22 that ejects its DNA after binding to the extracted phage receptor *Salmonella* LPS (Andres et al., 2010). These results confirm that the CA is the receptor for phage POP72. It is interesting to note that eight phages from the nine isolated phages including POP72 use CA as a receptor (Figure S4), implying a wide involvement of CA as a receptor among Pcc phages.

### Effect of Spontaneous Mutation in the CA Biosynthetic Gene on Phage POP72 Susceptibility

**Figure 4:**
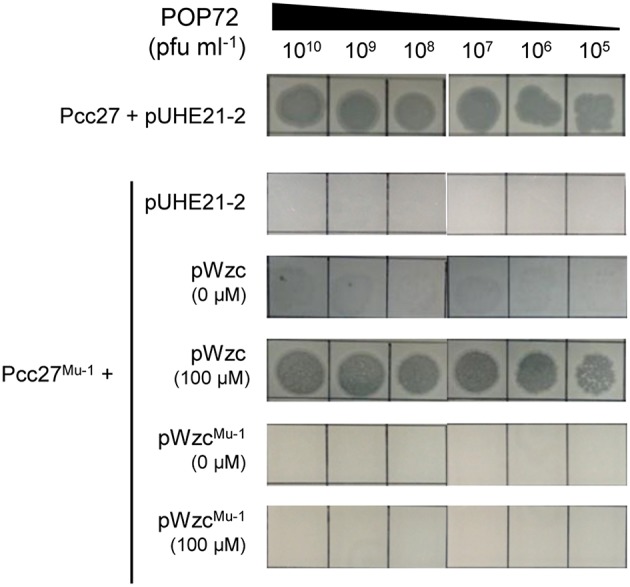
Spontaneous mutation in the *wzc* gene affects the susceptibility of Pcc27 to phage POP72. Phage spot assays with the WT Pcc27 and Pcc27^Mu−1^ possessing a point mutation in the *wzc* gene were conducted. The WT *wzc* gene (pWzc) or mutant *wzc* gene (pWzc^Mu−1^) was used to complement Pcc27^Mu−1^. IPTG concentrations are indicated in parentheses. One representative result of three independent experiments is shown.

Bacteria generally evade phage infections by modifying or eliminating the phage receptor (Labrie et al., 2010). To investigate whether a similar phenomenon could occur in Pcc, spontaneous POP72-resistant Pcc27 mutants were isolated through the high-titer overlay assay as previously described (Kim and Ryu, 2011). Eleven mutants were obtained, and the resistance against POP72 infection was verified by the spot assay (data not shown). PCR amplification and DNA sequencing revealed missense mutations within the putative *wzc* gene from the two spontaneous mutants (Figure S5). In particular, one POP72-resistant mutant, designated Pcc27^Mu−1^, contained a single base substitution at the 1081st nucleotide from the start codon of the putative *wzc* gene: guanine (G) was substituted by thymine (T). This substitution resulted in a replacement of valine (V) with phenylalanine (F) at amino acid residue 361 of the *wzc* gene product. A nucleotide adenine (A) was replaced with a cytosine (C) at the 1229th nucleotide from the start codon of putative *wzc* gene in another POP72-resistant mutant Pcc27^Mu−2^, resulting in a substitution from aspartic acid (D) to alanine (A) in amino acid residue 410 of the gene product. Notably, these substitutions occurred near or at a G-rich domain characterized by a highly conserved GNVR sequence motif in the putative tyrosine kinase Wzc, suggesting that the substituted residues (V and D) are important for the normal activity of Wzc. When the spontaneous mutant Pcc27^Mu−1^ was complemented with the WT *wzc* gene, POP72 formed clear plaques on the bacterial lawn similar to the WT level (Figure 4). In contrast, complementation with the mutant *wzc* gene (*wzc*^Mu−1^) did not restore the susceptibility to POP72 (Figure 4), indicating that the single point mutation can lead to POP72 resistance. These results suggest that the CA biosynthesis genes, at least the putative *wzc* gene, play a critical role in the POP72 infection, probably biosynthesizing the phage receptor. Unidentified mutations in the genes other than the *wzc* gene related to the CA biosynthesis might be responsible for the POP72-resistance in the other nine spontaneous mutants.

### Quantification of CA From Various Pcc27 Strains

**Figure 5:**
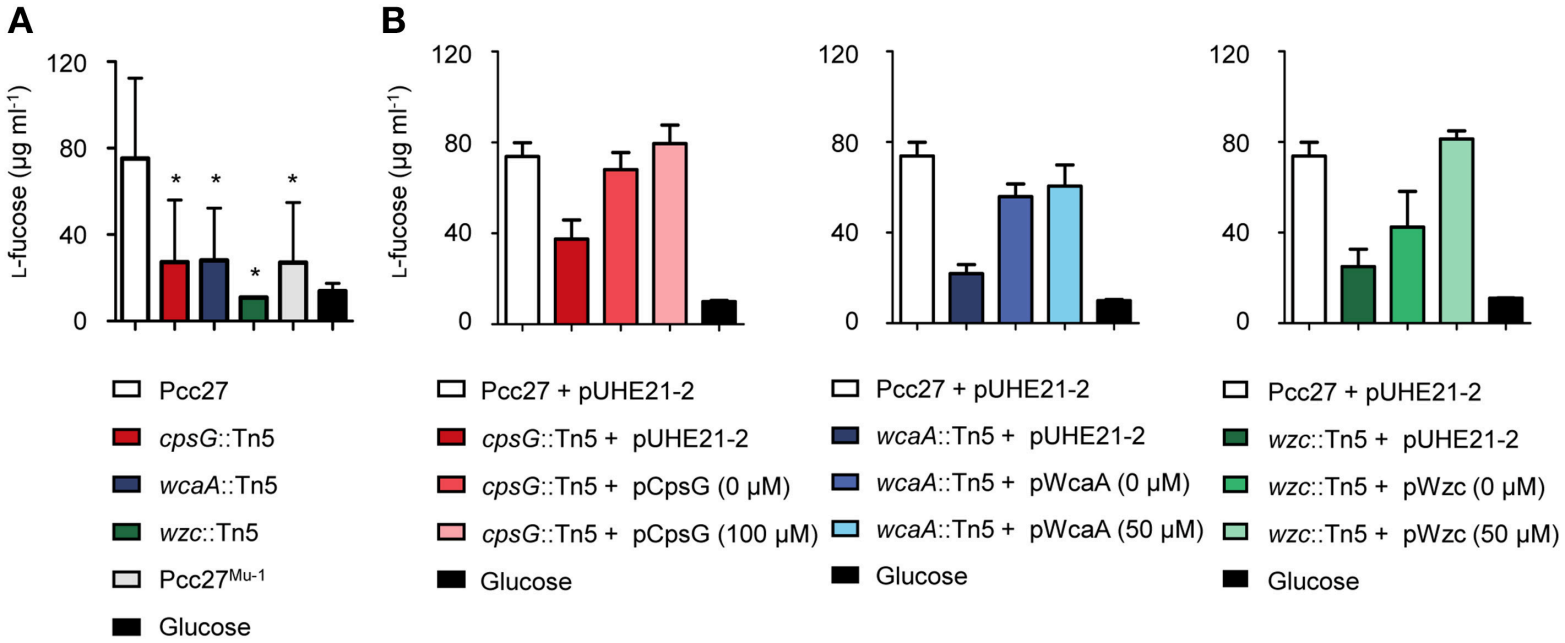
Determination of the total quantity of CA produced by Tn5 mutants and spontaneous POP72-resistant mutant **(A)** and each gene-complemented strain **(B)**. CA extracted from each Pcc27 culture was quantified by measuring l-fucose colorimetrically (details in the Materials and Methods). Glucose solution was used as a negative control. The means and standard deviations from three independent experiments are indicated with error bars. ^*^P < 0.05.

Based on these results, we hypothesized that CA is an important exopolysaccharide for the initial infection of phage POP72. To determine whether the phage-resistant mutants lost their CA from the bacterial cell surface, the CA was extracted and quantified from a stationary phase culture of each strain as described by Meredith et al. (2007) and Kim et al. (2015). As expected, both Tn5-inserted and spontaneous phage-resistant mutants produced approximately half the amounts of CA produced by the WT Pcc27 (Figure 5A). Complementation of the inactivated genes reversed not only the phage sensitivity (Figure 3A) but also the amounts of CA biosynthesized to the WT Pcc27 level (Figure 5B), further suggesting that CA production is required for phage POP72 infection.

### Protection of Pcc27 Infection in Chinese Cabbage by POP72

**Figure 6:**
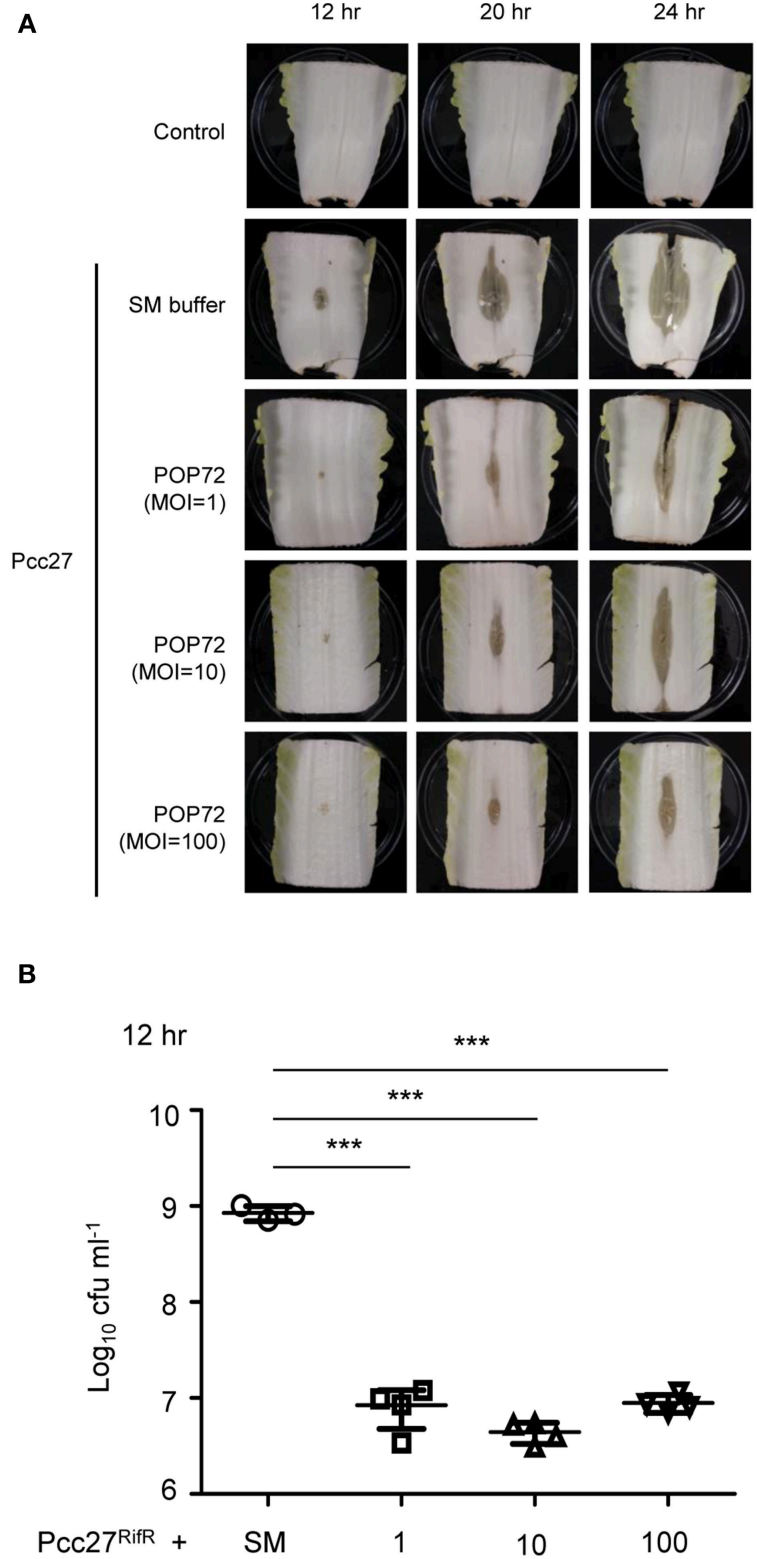
Retardation of soft rot disease development in Chinese cabbage by POP72 treatment. Artificially inoculated Chinese cabbages with Pcc27 were treated with or without an indicated MOI of POP72 and stored in a humid chamber. Disease symptoms **(A)** and the recovered bacterial cell numbers **(B)** were monitored at the time point(s) indicated. Non-inoculated Chinese cabbage was used as the negative control, and Pcc27-inoculated with SM buffer was used as non-phage control. One representative result of triplicated experiments is shown. ^***^P < 0.0001.

*Pectobacterium* spp. regulates the PCWDE expression under the control of quorum sensing using the *N*-acyl homoserine lactones (*N*-AHL) as signal molecules (Jones et al., 1993; Pollumaa et al., 2012). At high cell density, more than ~5.0 × 10^6^ CFU mL^−1^, this plant pathogen produces PCWDE and causes soft rot diseases on host crops and vegetables (Liu et al., 2008). To test the potential ability of POP72 as an antimicrobial agent against *Pectobacterium* spp. infection in vegetables, Chinese cabbage artificially inoculated with Pcc27 was treated with the POP72 phage. Rifampicin-resistant Pcc27 (Pcc27^RifR^) was used when it was necessary to quantify the Pcc cells recovered from Chinese cabbage. As expected, Pcc27, as well as Pcc27^RifR^, caused a prominent soft rot disease in Chinese cabbage when the initial spiked bacterial cell density was more than 10^6^ CFU mL^−1^ (data not shown). Pieces of Chinese cabbage (cut ~10 by 7 cm) inoculated with 10^7^ CFU mL^−1^ of Pcc27 started to show a typical soft rot of cabbage tissues after 12 h of storage at 28°C. In contrast, symptom development, as well as bacterial growth, was comparatively retarded up to 12 h by the POP72 treatment (Figures 6A,B). Although the softening of the cabbage tissues treated with POP72 started after 12 h of Pcc27 infection, the extent of the tissue softening was still less significant compared with the untreated cabbage. In addition, the progress of the symptoms was mitigated with the higher concentration of POP72 (Figure 6A). The recovered Pcc population was also reduced to a significant level by POP72 treatment at MOIs of 10 and 100 compared with the SM buffer-treated control (Figure 6B). These results indicate that phage POP72 has the potential to serve as an antimicrobial agent to control *Pectobacterium* spp. Further studies to elucidate the optimum phage treatment protocol to control Pcc in Chinese cabbage are required.

## Conclusion

In this study, we identified colonic acid (CA) as a novel phage receptor for phage POP72 through the screening of a Tn5 random mutant library of the host *P. carotovorum* subsp. *carotovorum* Pcc27 isolate. The specific binding moiety for the POP72 adsorption in the Pcc CA need to be further investigated to elucidate the details of the initial interaction between POP72 and Pcc. In addition, phage adsorption to the bacterial surface polysaccharides has been generally reported to be accompanied by endoglu- or endoglycosidase activity of the phage tails (Stirm et al., 1971; Born et al., 2014). Thus, whether similar enzymatic reactions were involved in the POP72 infection also needs to be investigated through further biochemical study.

Although functions of the extracellular polysaccharides are well characterized in other plant pathogens, roles of CA in growth, stress response, biofilm formation, or virulence have not been comprehensively studied in *Pectobacterium*. Capsular EPS of plant pathogens generally play protective roles against plant immune systems and plant-derived toxins and assist bacterial adherence to the plant tissue (Newman et al., 2007; Pfeilmeier et al., 2016), suggesting similar roles for CA in Pcc. CA might also be associated with the pathogenesis and virulence of Pcc, like amylovoran and levan in *E. amylovora* (Roach et al., 2013). Therefore, POP72-resistant Pcc strain, which cannot make CA, may have lower virulence.

POP72 delayed the development of soft rot disease caused by Pcc infection in the model Chinese cabbage up to 12 h, indicating the significant potential of POP72 as an antimicrobial agent for agricultural products. We are currently trying to isolate and characterize other Pcc phages that recognize phage receptors other than CA, using POP72-resistant mutants as host cells similar to the previous study (Kim et al., 2014). POP72 and these new phages can be applied simultaneously with the phage cocktail to more efficiently control the plant pathogen Pcc.

## Author Contributions

HK, MK, and SR conceived and designed the experiments. HK, MK, and JB performed the experiments and analyzed the data. HK and MK wrote the paper. J-AL and SH provided HK, MK, and JB with the *Pectobacterium carotovorum* strains. SR revised the manuscript.

### Conflict of Interest Statement

The authors declare that the research was conducted in the absence of any commercial or financial relationships that could be construed as a potential conflict of interest.
