# Supplementary material for: Colanic Acid Is a Novel Phage Receptor of Pectobacterium carotovorum subsp. carotovorum Phage POP72
Source: Front Microbiol. 2019 Feb 19;10:143. doi: 10.3389/fmicb.2019.00143 (PMC6390001; doi:10.3389/fmicb.2019.00143)
Supplement: Supplementary file 1 [file Table_1.pdf]

Supplementary material

Colanic Acid is a Novel Host Receptor of *Pectobacterium carotovorum* subsp. *carotovorum* Phage POP72

Hyeongsoon Kim[1, †], Minsik Kim[2, †], Jaewoo Bai[1], Jeong-A Lim[3], Sunggi Heu[4], and Sangryeol Ryu[1,5*]

[1]Department of Agricultural Biotechnology, Department of Food and Animal Biotechnology, Research Institute of Agriculture and Life Sciences, Seoul National University, Seoul 08826, Korea

[2]Department of Food and Nutrition, College of Human Ecology, Yonsei University, Seoul 03722, Korea

[3]Research Group of Food Safety, Korea Food Research Institute, Wanju-gun 55365, Korea

[4]Crop Cultivation and Environmental Research Division, National Institute of Crop Science, Suwon 16429, Korea

[5]Center for Food and Bioconvergence, Seoul National University, Seoul 08826, Korea

* Corresponding author.

Mailing address: Department of Food and Animal Biotechnology, Seoul National University, Seoul 08826, Korea

Phone: 82-2-880-4856. Fax: 82-2-873-5095. E-mail: sangryu@snu.ac.kr

[†]These authors contributed equally to this work.

Supplementary material

Supplementary Results

Morphological analysis of POP72

Phage POP72 was morphologically characterized using a transmission electron microscopic (TEM) analysis. POP72 has an icosahedral head and a short tail without a contractile sheath, suggesting that it belongs to the family *Podoviridae* of the order *Caudovirales*. The mean diameters of the isomeric head and the length of the short tail were approximately 60.09 ± 2.79 nm and 15.00 ± 3.80 nm, respectively (n = 22) (Supplementary Figure S1). Morphologically, POP72 resembles the bacteriophage T7 (head diameter: 60 nm, tail length: 23 nm) (Kemp et al., 2005).

Complete genome analysis of POP72

The phage POP72 genome was analyzed following 454 pyrosequencing. POP72 has 44,760 bp of double-stranded DNA with a GC content of 49.7% containing 55 putative open reading frames (ORFs) and no tRNAs (Supplementary Figure S2 and Supplementary Table S2). The National Center for Biotechnology Information (NCBI) database was utilized to predict a function of the protein from each ORF. Among 55 putative ORFs, 25 were expected to encode proteins of the following six specific functions: blocking of the host restriction, transcription regulation, DNA replication/modification, host lysis, structure and packaging, and additional functions. ORF006 encodes an S-adenosyl-L-methionine hydrolase that may play a role in overcoming the host restriction system upon phage infection (Siersma and Lederberg, 1970). ORF010 encodes a T7-like phage DNA-directed RNA polymerase that may be required to control the expression of the phage DNA polymerase, similar to the that of phage T7 (Kropinski et al., 2007). ORF013, ORF017, ORF018, ORF027, ORF028, and ORF031 encode DNA primase, DNA polymerase, HNH homing endonuclease, exonuclease,

endonuclease, and DNA ligase, respectively, for DNA replication and modification. They are followed by the ORFs for the phage structural proteins: major capsid protein and internal virion proteins encoded by ORF038, ORF041, and ORF043, respectively, and tail tubular protein A, tail tubular protein B and tail fiber protein encoded by ORF039, ORF040 and ORF044, respectively. The small and large subunit of the terminase required to package the replicated phage DNA into newly synthesized progenies are encoded by ORF046 and ORF047, respectively. ORF034 and ORF050 encode an acyl-CoA N-acyltransferase domain-containing protein and acyl-CoA acetyltransferase, respectively, but the functions of these proteins in phage infection/replication have not yet been specifically revealed.

In particular, ORF042, ORF045, ORF052, and ORF054 were predicted to encode proteins involved in the disruption of bacterial cells (Supplementary Figure S2). ORF042 encodes a putative peptidoglycan hydrolase that shares 57% identity with a gene product of *gp36* in *Salmonella* phage SP6 (Dobbins et al., 2004). A protein from ORF045 is a putative phage holin of the T7 family type II holin (Moak and Molineux, 2004). ORF052 encodes a peptidase M15A domain-containing protein that was predicted to be a muramoyl pentapeptide or carboxypeptidase (Scholl et al., 2004). A protein from ORF054 possesses an SGNH hydrolase domain at its N-terminus (116-301 residues), and it represents a catalytic triad and oxyanion hole similar to other SGNH hydrolase domain-containing proteins. The SGNH hydrolase domain was found in a diverse family of lipases and esterases in bacteria, and the activity of such enzymes derived from phages has been reported to be phage tail lysin (Li et al., 2016). Each of these enzymes is supposed to play a specific role to support the rapid lysis of Pcc cells by the phage. No genes known to be associated with lysogen formation were found, suggesting the lytic nature of the POP72.

Comparative genomic analysis was also conducted to elucidate the systemic similarity of

POP72 with other phages. Genome alignment using BLAST and ACT13 revealed that phage POP72 highly resembled *Pectobacterium carotovorum* phage PP1 (Lim et al., 2013). Although the whole genome of phage POP72 shares 99% nucleotide identity with the PP1 genome (Supplementary Figure S3), an additional 360-bp sequence flanked by ORF28 (endonuclease) and ORF29 (phosphoesterase) exists in POP72. Five more ORFs (ORF008, ORF011, ORF024, ORF026, and ORF051) enconding unknown proteins were also predicted from the POP72 genome compared to the PP1 genome. The other 48 ORFs of POP72 were highly homologous to those of phage PP1. Three of these proteins show single amino acid sequence differences: Ile at residue 248 of the putative DNA primase (ORF013), Asp at residue 82 of the putative tail tubular protein A (ORF039), and Ser at residue 666 of the putative tail tubular protein B (ORF40) in PP1 were substituted with Val, Ile, and Gly in POP72, respectively. However, the host ranges of POP72 and PP1were same (Supplementary Table S1), suggesting a close relationship between these two phages.

With the exception of phage PP1, phage phD2B that specifically infects the plant pathogen *Lelliottia* spp. is the phage most similar to POP72. Phage phD2B exhibited 81% sequence identity in the regions encoding the proteins for DNA replication/modification, structure and packaging, and host lysis (Nowicki et al., 2014). *Salmonella* virus SP6 (accession number NC_004831) and *Enterobacteria* phage UAB_Phi78 (accession number NC_020414.1) also have similarity with POP72 in their genome arrangement (Bardina et al., 2016).

Supplementary Figure

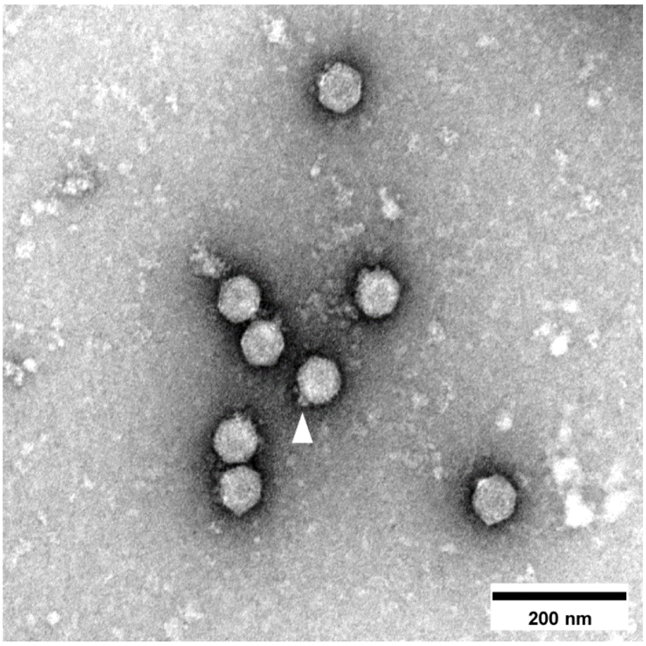

Figure S1. Transmission electron micrographs of phage POP72 particles negatively stained with 2% aqueous uranyl acetate (pH 4.0). The white arrow head indicates the tail shaft. Scale bar, 200 nm.

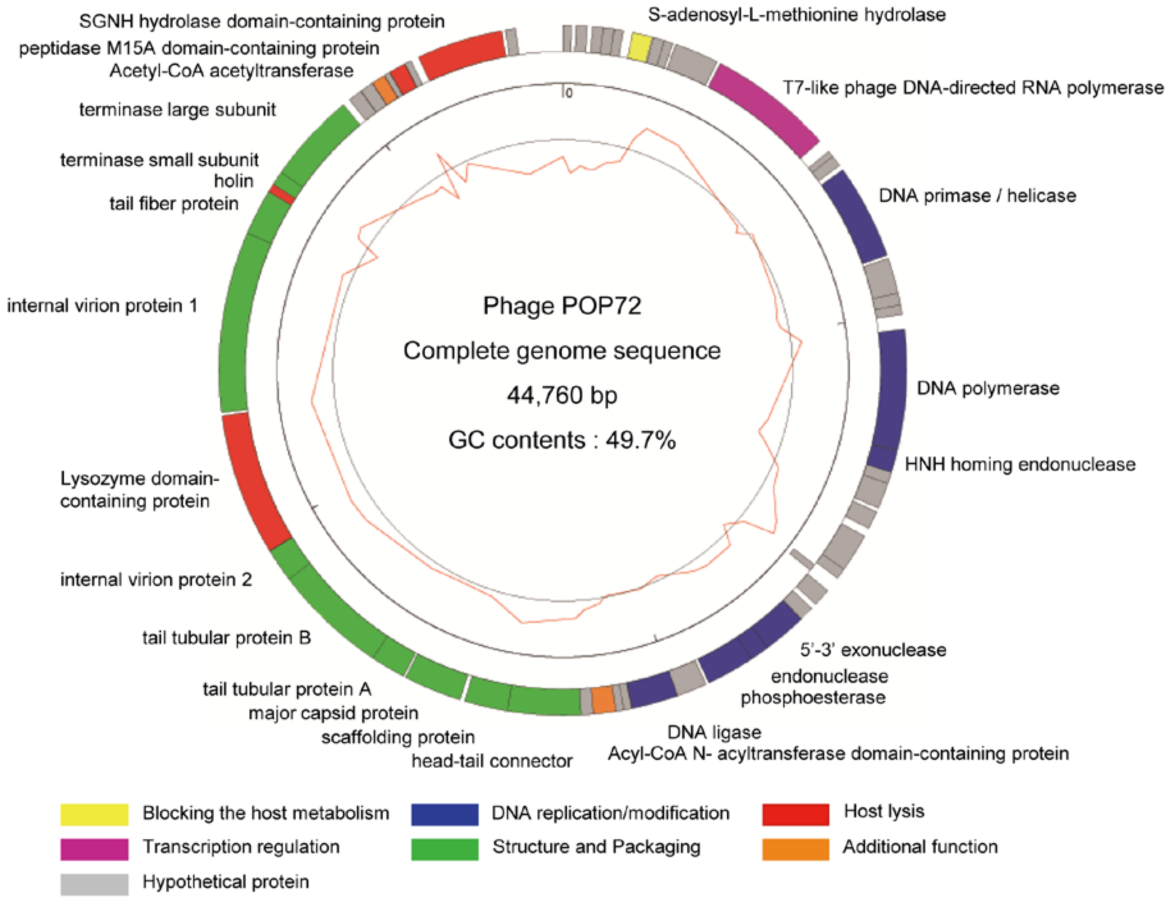

Figure S2. Whole genome map of POP72. The predicted ORFs with the corresponding gene products are indicated in color based on their functions. The inner orange line shows the %GC content of the phage genome.

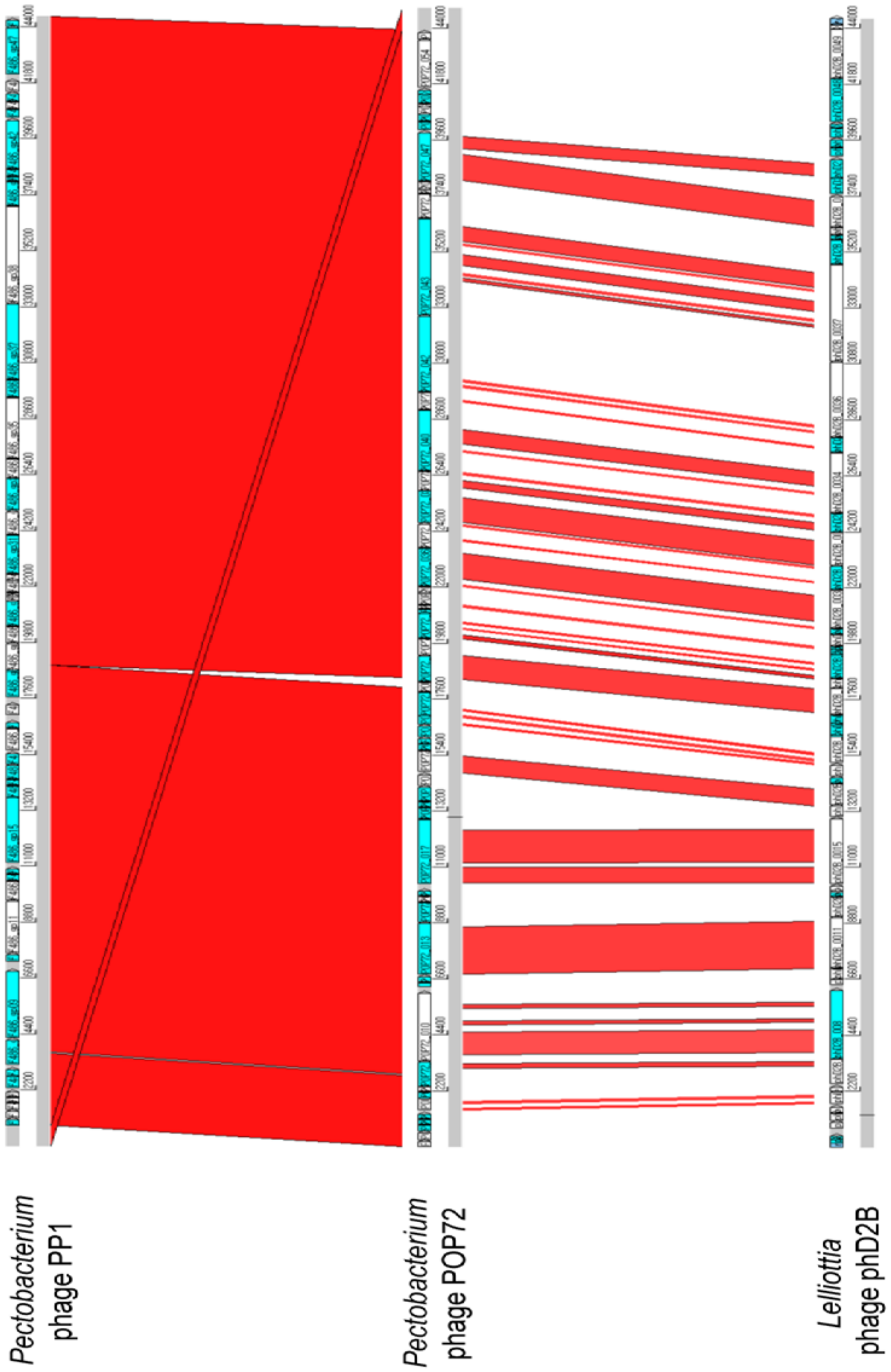

Figure S3. Comparative genomic analysis of phage POP72 with *Pectobacterium carotovorum* subsp. *carotovorum* phage PP1 and *Lelliottia* phage phD2B. The whole genome of the three phages was aligned and compared using the Artemis Comparison Tool 13. The homologous regions between the three phages are indicated in red.

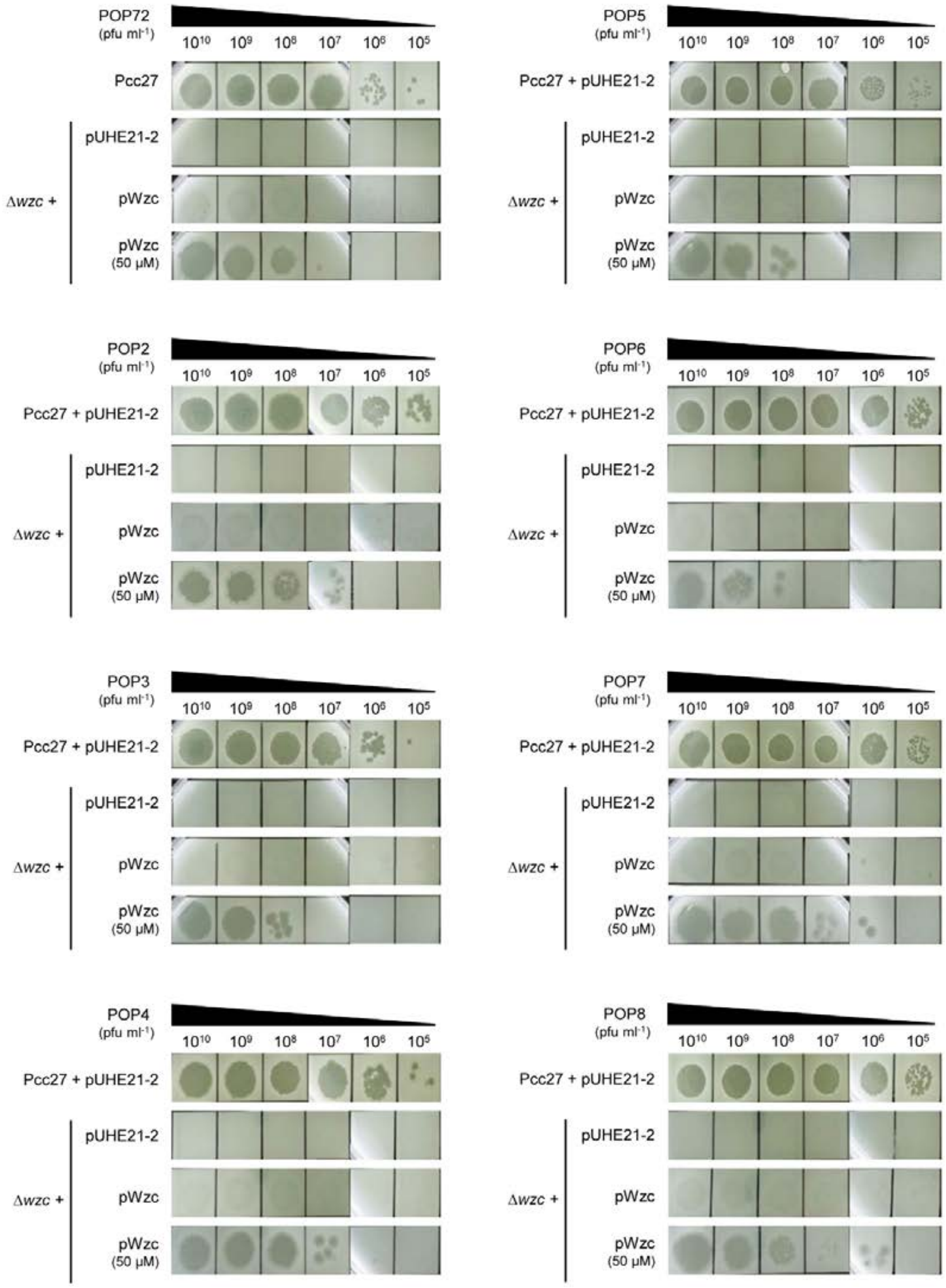

Figure S4. Determination of phage receptor for POP72 as well as other 7 phages in our collection. Complementation of the CA biosynthesis gene in an in-frame deletion mutant (Δ*wzc*) partially restored the phage susceptibility. The concentration of IPTG is indicated in parentheses. One representative of triplicate experiments are shown.

```
                                  20                    40                    60                    80
Pcc27      MAEKISVKTA ETNSDEIDLG RLLGTLLDNR WLIVGVTAVF TIIGILYATF ATPIYKADAL VQVEQNSGNA LLKDISSSLP
Pcc27Mu-1  .......... .......... .......... .......... .......... .......... .......... ..........
Pcc27Mu-2  .......... .......... .......... .......... .......... .......... .......... ..........

                                 100                   120                   140                   160
Pcc27      DSKPESAAEI EIIKSRMVVG KTVNDLSLSI VVQQKYFPIF GKGFARLMDE KPGRIAVSRL EVPKSWQDET IELRVLDAQT
Pcc27Mu-1  .......... .......... .......... .......... .......... .......... .......... ..........
Pcc27Mu-2  .......... .......... .......... .......... .......... .......... .......... ..........

                                 180                   200                   220                   240
Pcc27      YEIEAGDAVK FEGKVGQIAK HDNISLLVSD IQAEVGTVFR LQKLNSLTAI NNVLSKFIVA DKGKDTGVLA LSFEGEDPEL
Pcc27Mu-1  .......... .......... .......... .......... .......... .......... .......... ..........
Pcc27Mu-2  .......... .......... .......... .......... .......... .......... .......... ..........

                                 260                   280                   300                   320
Pcc27      TNKILNSISN NYLQQNVERK SEEAGKSLEF LKEQLPDVRL NLDQADDKLN TYRQENESVD LSLEAKAVLD TMVAIETQLN
Pcc27Mu-1  .......... .......... .......... .......... .......... .......... .......... ..........
Pcc27Mu-2  .......... .......... .......... .......... .......... .......... .......... ..........

                                 340                   360                   380                   400
Pcc27      ELTFKEAEVS KLYTKQHPTY RSLMEKRKTL EDEREKINKR VAAMPKTQQE ILRLMRDVNV GQEVYMQLLN KQQELSINKA
Pcc27Mu-1  .......... .......... .......... .......... F......... .......... .......... ..........
Pcc27Mu-2  .......... .......... .......... .......... .......... .......... .......... ..........

                                 420                   440                   460                   480
Pcc27      STVGNVRIID PSAIQLSPVK PKKVLVVLLA MLLGGMISTA FVVLRAVLHK GIESPEQLEE MGINVYASVP LSEWQQKKDR
Pcc27Mu-1  .......... .......... .......... .......... .......... .......... .......... ..........
Pcc27Mu-2  .........A .......... .......... .......... .......... .......... .......... ..........

                                 500                   520                   540                   560
Pcc27      VLLTRSNKSN TRSTELLAIG NPTDLAIEAI RSLRTSLHFA MMEAKNNVLM ISGASPAIGK TFISANLGAV ISQSGQRVLI
Pcc27Mu-1  .......... .......... .......... .......... .......... F......... .......... ..........
Pcc27Mu-2  .......... .......... .......... .......... .......... .......... .......... ..........

                                 580                   600                   620                   640
Pcc27      VDCDMRKGYA HELMGTQSVD GLSDILSGQI AVEKSIRKTA VDNMDFIPRG QIPPNPSELL MHSRFPEFIK WAAEHYDIVL
Pcc27Mu-1  .......... .......... .......... .......... .......... .......... .......... ..........
Pcc27Mu-2  .......... .......... .......... .......... .......... .......... .......... ..........

                                 660                   680                   700                   720
Pcc27      LDTPPILAVT DAAIISRHAG TSLLVARFEV NTLKEIEVSI RRFEQNGAEI KGVILNAIVK RAASYYGYGN YHYYTYDYKS
Pcc27Mu-1  .......... .......... .......... .......... .......... .......... .......... ..........
Pcc27Mu-2  .......... .......... .......... .......... .......... .......... .......... ..........

Pcc27      EKTN* 725
Pcc27Mu-1  ....* 725
Pcc27Mu-2  ....* 725
```

Figure S5. Multiple amino acid sequence alignment of Wzc from WT Pcc27 and spontaneous POP72-resistant mutants (Pcc27^{Mu-1}, Pcc27^{Mu-2}). Mismatched amino acid residues in spontaneous mutants are shaded in gray and matched residues are shown as dots. G-rich domain with highly conserved GNVR sequence motif in putative tyrosine kinase was represented with black bar.

Supplementary Table

Table S1. Host range of phage POP72[a]

Bacterial isolate	POP1	POP2	POP3	POP4	POP5	POP6	POP7	POP8	POP72	PP1
Pectobacterium carotovorum* subsp. *carotovorum										
Pcc3	-	+	+	+	+	+	+	+	+	+
Pcc4	-	+	+	+	+	+	+	+	+	+
Pcc8	-	I	I	I	I	I	I	I	+	+
Pcc9	-	I	I	I	I	I	I	I	+	+
Pcc10	-	I	I	I	I	I	I	I	+	+
Pcc11	-	-	-	-	-	-	-	-	+	+
Pcc12	-	-	-	-	-	-	-	-	+	+
Pcc13	-	-	-	-	-	-	-	-	+	+
Pcc14	-	-	-	-	-	-	-	-	+	+
Pcc15	-	-	-	-	-	-	-	-	+	+
Pcc17	-	-	-	-	-	-	-	-	+	+
Pcc21	-	I	I	I					-	-
Pcc22	-	+	+	+	+	+	+	+	+	+
Pcc27	-	+	+	+	+	+	+	+	+	+
Pcc29	-	+	+	+	+	+	+	+	+	+
Pcc96	+	-	-	-	-	-	-	-	-	-
Pcc97	-	+	+	+	+	+	+	+	+	+
Pcc101	-	-	-	-	-	-	-	-	+	+
Pcc102	-	-	-	-	-	-	-	-	+	+
E13	+	-	-	-	-	-	-	-	-	-
E17	-	+	+	+	+	+	+	+	+	+
E6	-	I	I	I	I	I	I	I	+	+
Pcc38	-	+	+	+	+	+	+	+	+	+
Pectobacterium carotovorum* subsp. *brasiliensis										
Pcc1	-	-	-	-	-	-	-	-	+	+
Pcc5	-	+	+	+	-	-	-	-	+	+
Pcc25	-	-							-	-
Pcc26	-	-							+	+
Pcc49	-	-							+	+
Pcc87	-	-							+	+
Pcc88	-	-							+	+
Pcc99	-	-							+	+
E10	-	I	I	I	I	I	I	I	+	+
E12	-	I	I	I	I	I	I	I	+	+
E3	-	I	I	I	I	I	I	I	+	+
E35	-	+	+	+	+	+	+	+	+	+
Pcc0001	-	I	I	I	I	I	I	I	+	+
Pcc0031	-	I	I	I	I	I	I	I	+	+

[a]+, *presence of plaques; -, absence of plaques; I, inhibition zone.*

Table S2. Predicted open reading frames (ORFs) of POP72 and predicted database matches

Locus_tag	ORF	Start	End	Predicted functions	BlastP (Best match)[a]	Identity (%)[b]
POP72_001	001	1	177	hypothetical protein	hypothetical protein PP1_001 [Pectobacterium phage PP1]	58/58 (100%)
POP72_002	002	271	510	hypothetical protein	hypothetical protein PP1_002 [Pectobacterium phage PP1]	79/79 (100%)
POP72_003	003	617	841	hypothetical protein	hypothetical protein PP1_003 [Pectobacterium phage PP1]	73/74 (99%)
POP72_004	004	841	1065	hypothetical protein	hypothetical protein PP1_004 [Pectobacterium phage PP1]	74/74 (100%)
POP72_005	005	1067	1261	hypothetical protein	hypothetical protein PP1_005 [Pectobacterium phage PP1]	64/64 (100%)
POP72_006	006	1461	1877	S-adenosyl-L-methionine hydrolase	putative S-adenosyl-L-methionine hydrolase [Pectobacterium phage PP1]	138/138 (100%)
POP72_007	007	1903	2136	hypothetical protein	hypothetical protein PP1_007 [Pectobacterium phage PP1]	77/77 (100%)
POP72_008	008	2136	2318	hypothetical protein	alpha-ketoacid dehydrogenase subunit beta [Quadrisphaera granulorum]	19/57 (33%)
POP72_009	009	2382	3311	hypothetical protein	hypothetical protein PP1_008 [Pectobacterium phage PP1]	309/309 (100%)
POP72_010	010	3389	6010	DNA-directed RNA polymerase	putative DNA-directed RNA polymerase [Pectobacterium phage PP1]	859/859 (100%)
POP72_011	011	6295	6426	hypothetical protein	hypothetical protein phD2B_009 [Lelliottia phage phD2B]	21/36 (58%)
POP72_012	012	6428	6658	hypothetical protein	hypothetical protein PP1_010 [Pectobacterium phage PP1]	76/76 (100%)
POP72_013	013	6774	8762	DNA primase/helicase	DNA primase [Pectobacterium phage PP1]	661/662 (100%)
POP72_014	014	8836	9579	hypothetical protein	hypothetical protein PP1_012 [Pectobacterium phage PP1]	247/247 (100%)
POP72_015	015	9579	9836	hypothetical protein	hypothetical protein PP1_013 [Pectobacterium phage PP1]	85/85 (100%)

Table S2. Continued

Locus_tag	ORF	Start	End	Predicted functions	BlastP (Best match)[a]	Identity (%)[b]
POP72_016	016	9829	10026	hypothetical protein	hypothetical protein PP1_014 [Pectobacterium phage PP1]	65/65 (100%)
POP72_017	017	10338	12851	DNA polymerase	putative DNA polymerase [Pectobacterium phage PP1]	837/837 (100%)
POP72_018	018	12865	13326	HNH homing endonuclease	HNH endonuclease [Pectobacterium phage PP1]	153/153 (100%)
POP72_019	019	13327	13569	hypothetical protein	hypothetical protein PP1_017 [Pectobacterium phage PP1]	80/80 (100%)
POP72_020	020	13569	14111	hypothetical protein	hypothetical protein PP1_018 [Pectobacterium phage PP1]	180/180 (100%)
POP72_021	021	14199	14576	hypothetical protein	hypothetical protein PP1_019 [Pectobacterium phage PP1]	125/125 (100%)
POP72_022	022	14762	15568	hypothetical protein	hypothetical protein PP1_020 [Pectobacterium phage PP1]	268/268 (100%)
POP72_023	023	15570	15791	hypothetical protein	hypothetical protein PP1_021 [Pectobacterium phage PP1]	73/73 (100%)
POP72_024	024	15864	16016	hypothetical protein	No match	-
POP72_025	025	16123	16494	hypothetical protein	hypothetical protein PP1_022 [Pectobacterium phage PP1]	123/123 (100%)
POP72_026	026	16619	16918	hypothetical protein	19 [Salmonella virus SP6]	42/102 (41%)
POP72_027	027	16930	17859	5'-3' exonuclease	putative 5'-3' exonuclease [Pectobacterium phage PP1]	309/309 (100%)
POP72_028	028	17844	18254	endonuclease	endonuclease [Proteus phage PM 93]	108/136 (79%)
POP72_029	029	18247	19254	phosphoesterase	hypothetical protein phD2B_0023 [Lelliottia phage phD2B]	296/333 (93%)
POP72_030	030	19325	19960	hypothetical protein	hypothetical protein PP1_025 [Pectobacterium phage PP1]	211/211 (100%)
POP72_031	031	19960	20934	DNA ligase	putative DNA ligase [Pectobacterium phage PP1]	324/324 (100%)
POP72_032	032	20945	21133	hypothetical protein	hypothetical protein PP1_027 [Pectobacterium phage PP1]	62/62 (100%)
POP72_033	033	21108	21278	hypothetical protein	hypothetical protein PP1_028 [Pectobacterium phage PP1]	56/56 (100%)

Table S2. Continued

Locus_tag	ORF	Start	End	Predicted functions	BlastP (Best match)[a]	Identity (%)[b]
POP72_034	034	21275	21760	acyl-CoA N-acyltransferase domain-containing protein	acyl-CoA N-acyltransferase domain-containing protein [Pectobacterium phage PP1]	161/161 (100%)
POP72_035	035	21747	22010	hypothetical protein	hypothetical protein PP1_030 [Pectobacterium phage PP1]	87/87 (100%)
POP72_036	036	22007	23533	head-tail connector	putative head-tail connector [Pectobacterium phage PP1]	508/508 (100%)
POP72_037	037	23533	24441	scaffolding protein	putative scaffolding protein [Pectobacterium phage PP1]	302/302 (100%)
POP72_038	038	24545	25762	major capsid protein	major capsid protein [Pectobacterium phage PP1]	405/405 (100%)
POP72_039	039	25815	26552	tail tubular protein A	putative tail tubular protein A [Pectobacterium phage PP1]	244/245 (99%)
POP72_040	040	26552	28942	tail tubular protein B	putative tail tubular protein B [Pectobacterium phage PP1]	795/796 (99%)
POP72_041	041	28945	29643	internal virion protein 2	putative internal virion protein 2 [Pectobacterium phage PP1]	232/232 (100%)
POP72_042	042	29653	32628	lysozyme domain-containing protein	lysozyme domain-containing protein [Pectobacterium phage PP1]	991/991 (100%)
POP72_043	043	32697	36485	internal virion protein 1	putative internal virion protein 1 [Pectobacterium phage PP1]	1262/1262 (100%)
POP72_044	044	36485	37453	tail fiber protein	putative tail fiber protein [Pectobacterium phage PP1]	322/322 (100%)
POP72_045	045	37450	37632	holin	putative holin [Pectobacterium phage PP1]	60/60 (100%)
POP72_046	046	37629	37928	terminase small subunit	putative terminase small subunit [Pectobacterium phage PP1]	99/99 (100%)
POP72_047	047	37928	39826	terminase large subunit	putative terminase large subunit [Pectobacterium phage PP1]	632/632 (100%)
POP72_048	048	39992	40282	hypothetical protein	hypothetical protein PP1_043 [Pectobacterium phage PP1]	96/96 (100%)
POP72_049	049	40292	40570	hypothetical protein	hypothetical protein PP1_044 [Pectobacterium phage PP1]	92/92 (100%)
POP72_050	050	40583	40864	acetyl-CoA acetyltransferase	hypothetical protein PP1_045 [Pectobacterium phage PP1]	93/93 (100%)

Table S2. Continued

Locus_tag	ORF	Start	End	Predicted functions	BlastP (Best match)[a]	Identity (%)[b]
POP72_051	051	40876	40992	hypothetical protein	hypothetical protein [Pseudoalteromonas luteoviolacea]	16/36 (44%)
POP72_052	052	41004	41351	M15A domain-containing protein	peptidase M15A domain-containing protein [Pectobacterium phage PP1]	115/115 (100%)
POP72_053	053	41361	41498	hypothetical protein	hypothetical protein phD2B_0047 [Lelliottia phage phD2B]	35/44 (79%)
POP72_054	054	41676	43478	SGNH hydrolase domain-containing protein	SGNH hydrolase domain-containing protein [Pectobacterium phage PP1]	600/600 (100%)
POP72_055	055	43560	43769	hypothetical protein	hypothetical protein PP1_048 [Pectobacterium phage PP1]	69/69 (100%)

a,The best matched homologs in phage POP72 are included.
b,The percentage identity is calculated based on the number of identical amino acid residues (numerator) over the number of compared residues (denominator) and shown in parentheses.

Supplementary material

Reference

Bardina, C., Colom, J., Spricigo, D.A., Otero, J., Sánchez-Osuna, M., Cortés, P., and Llagostera, M. (2016). Genomics of three new bacteriophages useful in the biocontrol of *Salmonella*. *Front Microbiol* 7**,** 545.

Dobbins, A.T., George, M., Basham, D.A., Ford, M.E., Houtz, J.M., Pedulla, M.L., Lawrence, J.G., Hatfull, G.F., and Hendrix, R.W. (2004). Complete genomic sequence of the virulent *Salmonella* bacteriophage SP6. *Journal of Bacteriology* 186**,** 1933-1944.

Kemp, P., Garcia, L.R., and Molineux, I.J. (2005). Changes in bacteriophage T7 virion structure at the initiation of infection. *Virology* 340**,** 307-317.

Kropinski, A.M., Sulakvelidze, A., Konczy, P., and Poppe, C. (2007). "*Salmonella* phages and prophages—genomics and practical aspects," in *Salmonella phages and prophages—genomics and practical aspects*. Springer), 133-175.

Li, X., Koç, C., Kühner, P., Stierhof, Y.-D., Krismer, B., Enright, M.C., Penadés, J.R., Wolz, C., Stehle, T., and Cambillau, C. (2016). An essential role for the baseplate protein Gp45 in phage adsorption to *Staphylococcus aureus*. *Sci Rep.* 6**,** 26455.

Lim, J.A., Jee, S., Lee, D.H., Roh, E., Jung, K., Oh, C., and Heu, S. (2013). Biocontrol of *Pectobacterium carotovorum* subsp *carotovorum* Using Bacteriophage PP1. *Journal of Microbiology and Biotechnology* 23**,** 1147-1153.

Moak, M., and Molineux, I.J. (2004). Peptidoglycan hydrolytic activities associated with bacteriophage virions. *Molecular Microbiology* 51**,** 1169-1183.

Nowicki, G., Barylski, J., Kujawa, N., and Gozdzicka-Jozefiak, A. (2014). Complete Genome Sequence of *Lelliottia* Podophage phD2B. *Genome Announc* 2.

Scholl, D., Kieleczawa, J., Kemp, P., Rush, J., Richardson, C.C., Merril, C., Adhya, S., and

Molineux, I.J. (2004). Genomic analysis of bacteriophages SP6 and K1-5, an estranged subgroup of the T7 supergroup. *Journal of Molecular Biology* 335**,** 1151-1171.

Siersma, P., and Lederberg, S. (1970). In Vivo Suppression of Coding Associated with Bacteriophage-Induced S-Adenosylmethionine Hydrolase. *Journal of bacteriology* 101**,** 398-407.
